# End-To-End Approximation of Cut Arteries

**Published:** 1897-02-06

**Authors:** 


### Medical Progress and Hospital Clinics.

[*The Editor will be glad to receive offers of co-operation and contributions from members of the profession. All letters should be addressed to* The Editor, at the Office, 28 & 29, Southampton Street, Strand, London, W.C.]

### END-TO-END APPROXIMATION OF CUT ARTERIES.

Those who profess to think that there is no scope left for the inventor, that all has been done, and that the world has been so exploited that the genius of to-day has no chance for the display of his originality, may be recommended to study the careful account given in the *New York Medical Record* for January 16th of a series of investigations as to the resection and end-to-end approximation of wounded arteries, recently made by Dr. J. B. Murphy, of Chicago.

Attempts have many times been made to close the openings in wounded arteries by means of suture, and in some cases with success. Indeed, a case is reported by Broca, in 1762, in which suture of a longitudinal incision of an artery was successfully made. In 1889 Bruci sutured six longitudinal incisions in arteries of dogs, and was successful in four; and Muscatello successfully sutured a one-third division of the abdominal aorta in a dog. Again, in April, 1895, Von Zoege-Manteuffel made a successful lateral suture of the wall of the common femoral artery, which he had accidentally wounded while operating on an arterio-venous aneurism in Scarpa's triangle. Wounded veins have often been closed by suture, and this is now an accepted surgical procedure.

What Dr. Murphy has done, however, is to resect the injured piece of the artery, to invaginate the proximal within the distal end, to fix the parts in their new position by suture, and to retain the patency of the lumen of the artery. No one can deny that this is a brilliant result, and the scientific interest of the matter is vastly increased by the fact that it is not a mere accidental success, but is the outcome of a series of carefully-conducted experiments planned and carried out with the definite intention of deciding on the possibility, and if possible on the best method, of producing an end-to-end junction of an artery.

Thirty-four separate experiments on animals are described, giving the modes of suture which were adopted, and the results. As the outcome of these experiments, Murphy has formulated a certain technique of arterial suture, which we need not here describe. We would, however, draw attention to the sort of cases in which the suture of arteries may probably come into vogue as a recognised operation in surgery. One of the first of these is the instance of accidental wounds inflicted on arteries during operations. In these, as in all cases, the desirability of suturing rather than ligaturing an artery must depend upon its individual importance to the vitality of the tissues on the distal side of the injury. Suture will probably be always more risky than immediate ligature, and where there are opportunities for the establishment of collateral circulation there will be no excuse for running any risk for the sake of the chance of maintaining patency of the vessel. Nor should such an operation be attempted where the artery is small, for any meddling with an artery always results in a narrowing of its lumen; it is only in large arteries, having a considerable lumen to begin with, that such an operation has any chance of success.

But in regard to large vessels, so circumstanced that interference with the circulation through them would be likely to lead to serious results, if a wound should take place during an operation, Murphy would advise immediate suture if less than two-thirds of the circumference of the vessel be involved; while if the mischief extend further than that, he would advise that the division should be made complete, and the method of invagination which he describes be used for the approximation of the divided ends, care being taken that the invaginating sutures do not penetrate the tunica intima of the invaginated portion. The sheath should be carefully sutured over the artery, as it gives additional support.

Next there are cases of injury from stab or bullet wounds, and then, resulting from such wounds, there are what Dr. Murphy describes as "the best variety of cases for arterial suture," viz., traumatic aneurisms of long standing. In these "the opening in the artery is usually small, the arterial wall is healthy, and a sufficient quantity of aneurismal stump may be obtained to produce a firm line of approximation." Sacculated, fusiform, and arterio-venous aneurisms are also included in Dr. Murphy's list.

Two cases are given by Dr. Murphy in which he has used suture in injuries of the blood vessels. The first was a bullet wound of the upper part of the thigh, in which there was a perforation of the internal sapheous vein and also of the femoral vein, in each case both the anterior and posterior surfaces having been traversed. The femoral artery had a fragment of tissue torn off the side of its sheath, but the vessel wall was not injured. The wounds in the veins were sutured, but unfortunately suppuration took place, a large swelling formed which was found to contain blood-clot, and the femoral artery became eroded and had to be tied above and below, after which the patient did well. What happened to the vein is not known. It was not located at the second operation, but there was no disturbance to the circulation of the limb. In the second case a bullet had entered Scarpa's triangle below Poupart's ligament, and a fortnight later, although there was no tumour, a loud bruit could be heard, even when the ear was held six inches from the thigh. On cutting down upon the artery it was found to have been traversed by the bullet and to have an aneurismal pocket both before it and behind. One eighth of an inch of the arterial wall on the outside of the opening remained while on the inner side only a band of one-sixteenth of an inch of adventitia was intact. A large hole had also been made in the vein, the hæmorrhage from which was very difficult to control. The vein was first sutured, after which the half-inch of the artery containing the bullet-hole was excised, and the cut ends approximated by invagination. On removing the clamps not a drop of blood escaped at the line of suture, and pulsation was immediately restored in the artery below the line of approximation, and could be felt feebly in the posterior tibial and dorsalis pedis. During the after progress of the case there was no œdema of the leg and no pain. The circulation was good, continuously from the time of the operation, and pulsation could be felt in the dorsalis pedis four days after its performance. The case is one in regard to which Dr. Murphy deserves most hearty congratulations.

